# Abnormal expressions of PURPL, miR-363-3p and ADAM10 predicted poor prognosis for patients with ovarian serous cystadenocarcinoma

**DOI:** 10.7150/jca.87405

**Published:** 2023-09-11

**Authors:** Ruitao Zhang, Xueying Guo, Limin Zhao, Tingting He, Wei Feng, Shumin Ren

**Affiliations:** Department of Gynecology, First Affiliated Hospital, Zhengzhou University, NO.1 East Jianshe Road, Erqi District, Zhengzhou, Henan, 450052, P.R. China.

**Keywords:** biomedical database, ovarian serous cystadenocarcinoma, PURPL, miR-363-3p, ADAM10, prognosis

## Abstract

**Objective:** This study aimed to elucidate the prognostic implications of deviant expressions of long non-coding RNA (lncRNA) p53 upregulated regulator of p53 levels (PURPL), microRNA-363-3p (miR-363-3p), and ADAM metallopeptidase domain 10 (ADAM10) in patients diagnosed with ovarian serous cystadenocarcinoma (OSC).

**Methods:** To predict and refine the targeted miRNAs and downstream target genes for PURPL, we utilized open medical databases. Through the employment of real-time RT-PCR, we conducted tissue analysis to discern the expressions of PURPL, miR-363-3p, and ADAM10 in both OSC and control tissues. The pathological correlations in the clinic and the prognostic implications of deviant expressions of PURPL, miR-363-3p, and ADAM10 in OSC patients were analyzed independently.

**Results:** Database inquiries revealed that PURPL might target miR-363-3p, and in turn, miR-363-3p could target ADAM10. Differential expression of PURPL, miR-363-3p, and ADAM10 was observed between OSC and paired tissues. The premature version of miR-363-3p, miR-363, correlated with overall survival (OS), while ADAM10 corresponded with progression-free survival (PFS) in ovarian cancer patients. Tissue detection displayed significantly elevated expressions of PURPL and ADAM10, and conspicuously diminished expressions of miR-363-3p in OSC tissues compared to the control tissues (P<0.05). A negative correlation was observed between the expressions of PURPL and miR-363-3p, and miR-363-3p and ADAM10, while a positive correlation was found between PURPL and ADAM10 in different ovarian tissues (P<0.05). In OSC tissues, upregulation of PURPL was associated with an advanced clinical stage, TP53 mutation, and lymph node metastasis (P<0.05), downregulation of miR-363-3p was associated with a more advanced clinical stage and lymph node metastasis (P<0.05), and overexpression of ADAM10 correlated with a more advanced FIGO stage. High expressions of PURPL and ADAM10, and low expression of miR-363-3p, were linked with poor PFS and OS in OSC patients, respectively (P<0.05). In addition, OSC patients with elevated PURPL and reduced miR-363-3p, patients with elevated PURPL and ADAM10, and patients with reduced miR-363-3p and elevated ADAM10 also demonstrated worse PFS and OS, respectively (P<0.05).

**Conclusions:** The anomalous expressions of PURPL, miR-363-3p, and ADAM10 might contribute to the pathogenesis of OSC via up-down stream regulation, and these abnormal expressions could serve as potential prognostic indicators for OSC patients.

## Introduction

Long non-coding RNAs (lncRNAs) have been substantiated to function as miRNA sponges, binding to target miRNAs as competitive endogenous RNAs to prevent degradation and functional inhibition of their downstream target genes. As a result, they play a crucial role in the genesis and progression of numerous malignant tumors, including ovarian cancer 1-4. Thus, lncRNAs hold promising potential as therapeutic targets and molecular markers for prognostic monitoring in various malignant tumors 5-9. Our preceding research illustrated that elevated expressions of long non-coding RNA LINC01021 (officially known as p53 upregulated regulator of p53 level, abbreviated as PURPL) were present in epithelial ovarian cancer tissues. This implies that PURPL could be implicated in the initiation and progression of ovarian cancer, and could serve as a prognostic indicator for adverse outcomes in ovarian cancer 10.

To delve further into the molecular mechanism by which PURPL contributes to the pathogenesis of ovarian serous cystadenocarcinoma (OSC), this study extracted information from open medical databases to identify miRNAs targeted by PURPL and downstream genes based on the regulatory interplay between lncRNA and miRNA. Guided by this database-derived information, we sought to evaluate the expressions of PURPL, miR-363-3p, and ADAM10 in normal ovarian tissues, ovarian serous cystadenoma, and OSC tissues using the real-time RT-PCR method. We aimed to establish the correlations between the expressions of these three factors, analyze the associations between the expressions of these factors and the clinicopathologic parameters of OSC, and explore their prognostic significance in OSC.

## Methods

### Biomedical database filter

Among the biomedical databases, LncBase Predicted 11, miRcode (http://www.mircode.org/), and ENCORI 12 were employed to predict the target miRNAs for PURPL. For the target miRNA, miRGator (http://mirgator.kobic.re.kr/) was used to delineate the differential expression between OSC and paired tissues. Kaplan-Meier Plotter 13 and OncomiR 14 were utilized to identify associations with survival in OSC patients. Downstream target genes of the intended miRNA were retrieved from TargetScan 15, microT-CDS (https://diana.e-ce.uth.gr/tools), miRDB 16, miRWalk (http://mirwalk.umm.uni-heidelberg.de/), and UALCAN 17. The differentially expressed target gene between OSC and paired tissues, which was also associated with the survival of patients with OSC, was isolated using Kaplan-Meier Plotter and UALCAN.

### Tissue specimens

This study serves as a continuation of our previously published work 10,18. Tissue samples consisting of normal ovarian tissues, serous cystadenoma tissues, and OSC tissues utilized in prior research were extracted and integrated into the current study specimens. Tissue sample collection commenced in October 2012 and concluded at the end of December 2017, with follow-up extending until December 2019. The study incorporated non-pregnant women requiring surgical intervention for gynecological disorders, with no prior history of gynecological malignancies, no preceding chemotherapy or radiation therapy, and without severe complications or malignancies involving other organs. Patients not meeting the aforementioned criteria were excluded from the study.

The study comprised a total of 30 samples of normal ovarian tissues, 30 samples of ovarian serous cystadenoma tissues, and 81 samples of primary OSC tissues. Patients of three groups ranged in age from 18 to 74, 24 to 72, and 28 to 74. There were no obvious differences in the data of age and BMI among three groups. The detailed clinical parameters of OSC patients were listed in Table 2. All the tissue samples were collected with the informed consent of the patients, in compliance with the ethical approval granted by the Ethics Committee of the First Affiliated Hospital of Zhengzhou University (NO. 2023-KY-0380-002).

### Real time RT-PCR

The procedure, reagent system, reaction conditions, and quality control for real time RT-PCR were executed in line with our previous study 18. PCR primers were synthesized by Shanghai Sangon Biotech Co., Ltd, and are detailed in Table 1. The relative expression values obtained through real time RT-PCR were presented utilizing the 2^-ΔΔCt^ method.

### Survival analysis

Patient follow-up commenced in October 2012 upon enrollment and confirmation of primary OSC by postoperative pathology, and extended until December 2019. The 81 enrolled OSC patients were followed for durations ranging from 10 to 81 months, with a median follow-up duration of 32 months. At the end of the follow-up period, 41 patients were alive and 40 had deceased. This study noted endpoint events as patient death and relapse-free survival. Overall survival (OS) time was characterized as the duration from the initiation of follow-up to the time of patient death attributable to the tumor or the last follow-up date. Progression-free survival (PFS) time was defined as the period from the start of follow-up to the time of patient recurrence due to the tumor or the last follow-up date. In instances where patients did not succumb or relapse at the end of the follow-up, these were categorized as truncation events and excluded from statistical calculations.

### Statistical Analysis

Average values are expressed as mean ± standard deviation. Data analysis was performed utilizing SPSS23.0 and GraphPad Prism 9 software packages. One-way ANOVA and Student's T-test were employed to examine expression differences. The Spearman correlation test was applied for expression interrelationship analysis. Chi-square test was utilized for assessing correlations with clinicopathologic characteristics. The Log-rank test was employed to analyze the impact of aberrant expressions on the survival of OSC patients, and the Kaplan-Meier method was used for the construction of survival curves. A P-value less than 0.05 was deemed statistically significant.

## Results

### High expression of LncRNA PURPL was correlated with poor patient survival in biomedical databases

**Figure 1:**
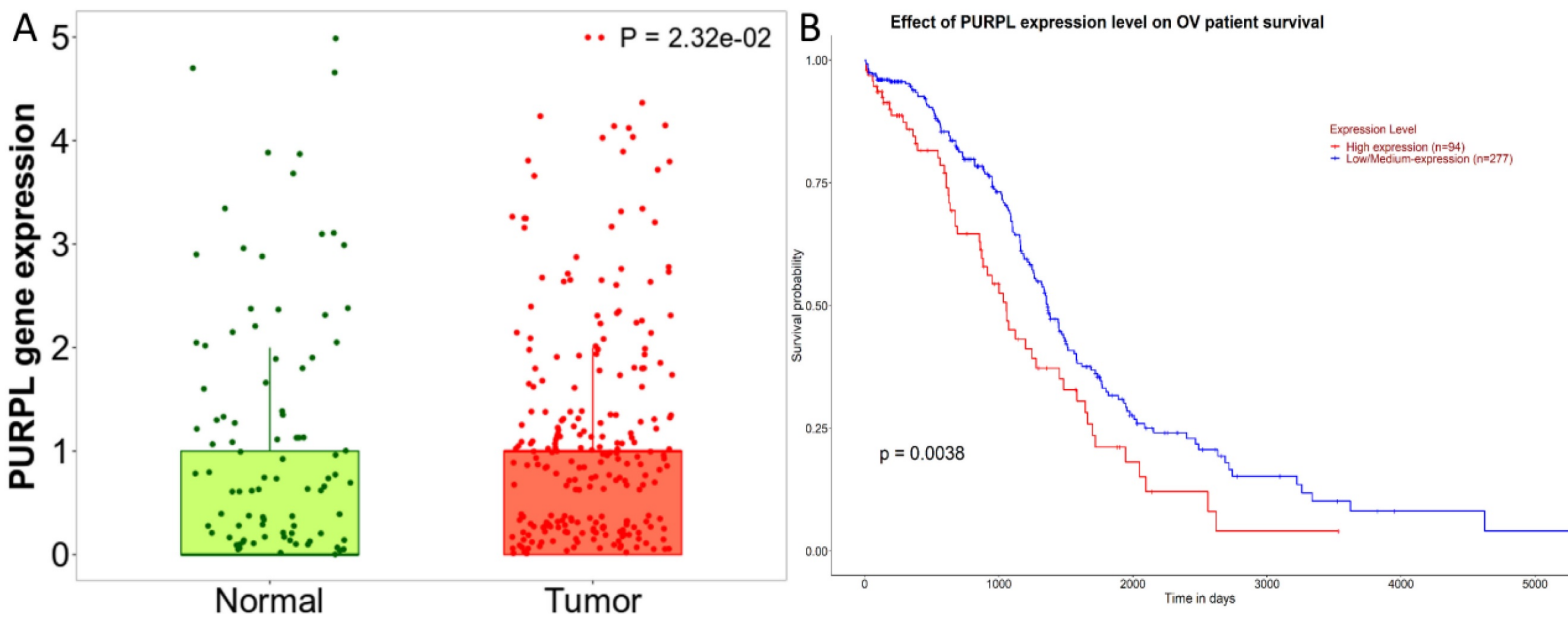
PURPL Expression Levels in OSC and the Correlation with Patient Survival (A. Comparative visualization of PURPL expression in OSC and matched normal ovarian tissues, data derived from the Kaplan-Meier Plotter database; B. The relationship between elevated PURPL expression and poor OSC patient survival as indicated by the UALCAN database.)

A detailed analysis for PURPL in OSC tissues versus normal samples from non-cancerous patients and additional pediatric tissues is provided by the Kaplan-Meier Plotter subset TN-plot, employing RNA-Seq based data. The PURPL level was elevated in 374 OSC tissue samples relative to 133 normal ovarian tissue samples (Figure 1A). The UALCAN subset TCGA lncRNA data indicated a poorer survival profile for OSC patients exhibiting high PURPL expression (Figure 1B). These results align with our previous ovarian cancer study 10.

### Low expression of miR-363-3p was correlated with poor patient survival in biomedical databases

**Figure 2:**
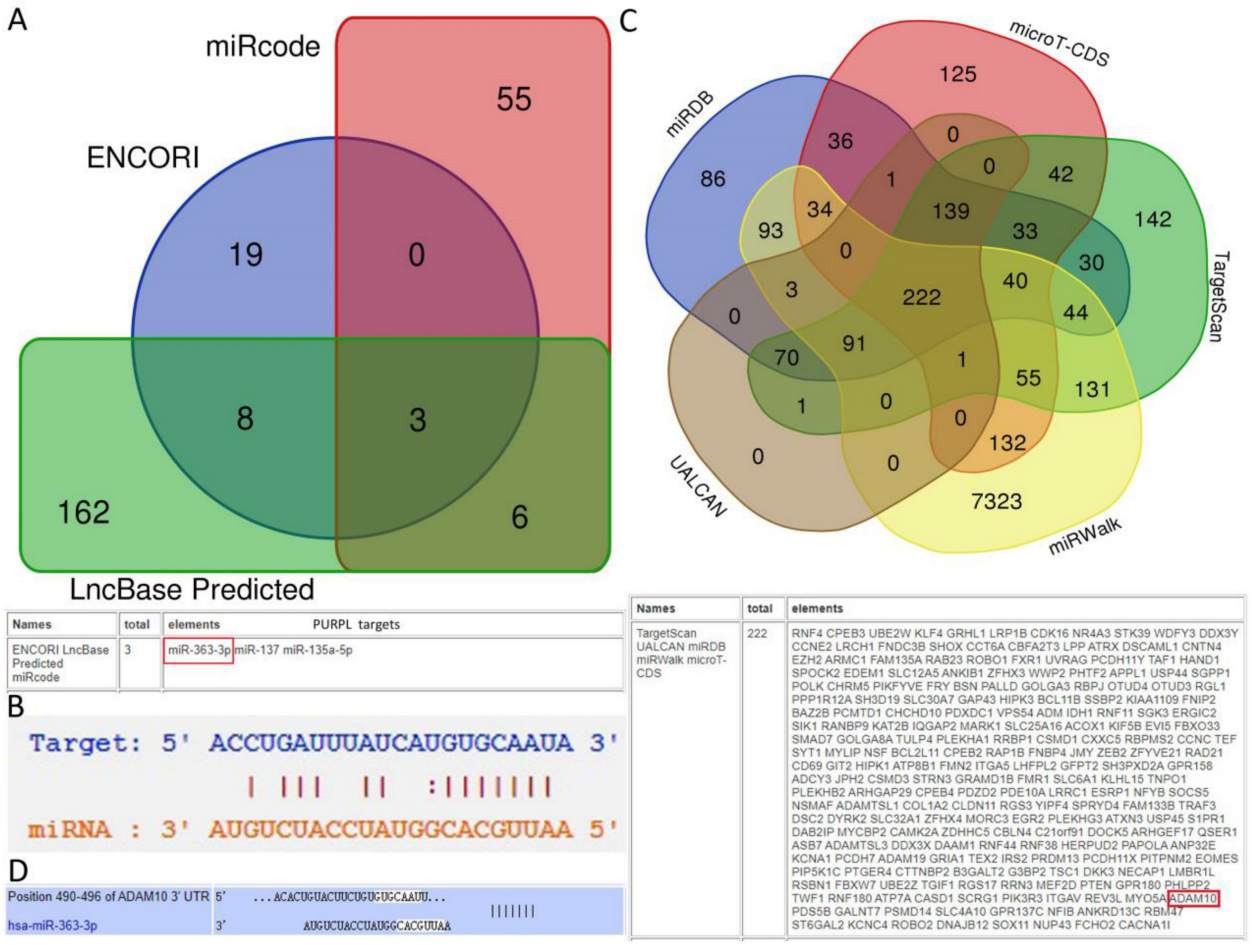
Databases Cross-prediction of target miRNAs for PURPL and target mRNAs for miR-363-3p (A. A Venn diagram depicting candidate downstream miRNAs for PURPL, as identified by the ENCORI, miRcode, and LncBase Predicted databases; B. Potential interaction sites between PURPL and miR-363-3p as listed in the ENCORI database; C. A Venn diagram showcasing downstream target genes for miR-363-3p, as predicted by the TargetScan, microT-CDS, miRDB, miRWalk, and UALCAN databases; D. Potential interaction sites between miR-363-3p and the ADAM10 gene as listed in the TargetScan database.)

**Figure 3:**
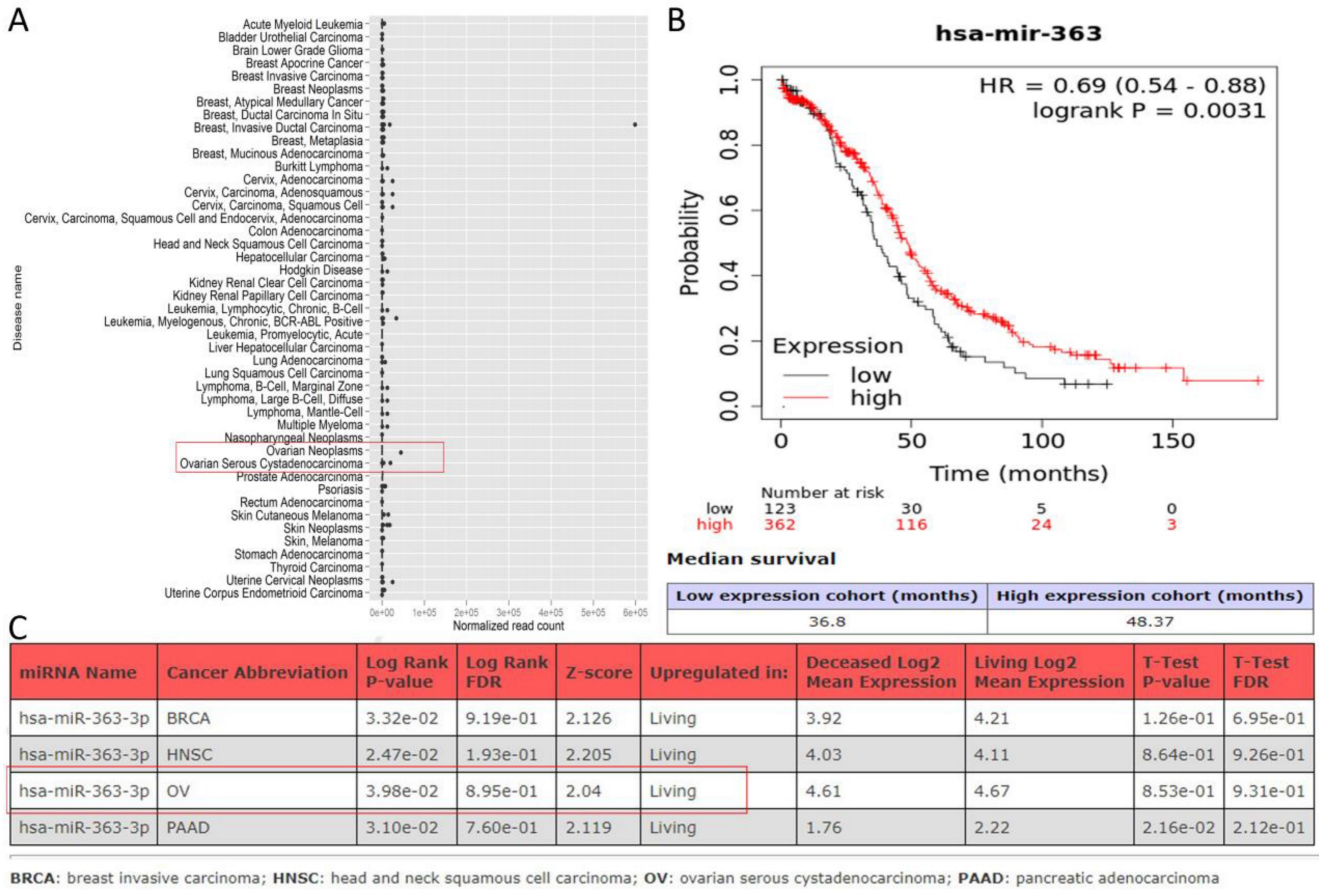
Expression Levels of miR-363-3p in OSC and the Impact on Patient Survival (A. Comparison of miR-363-3p expression levels in OSC and corresponding ovarian neoplasms, data sourced from the miRGator database; B. Association between low miR-363 expression and poorer Overall Survival (OS) in ovarian cancer, as illustrated by the Kaplan-Meier Plotter database; C. Correlation between upregulation of miR-363-3p and improved survival outcomes in OSC, as presented in the OncomiR database.)

Predictions from LncBase, miRcode, and ENCORI databases suggest that PURPL could bind to the 3 'UTR region of miR-363-3p (Figure 2A-B). High-throughput sequencing results obtained from miRGator demonstrated a significantly lower abundance of miR-363-3p in OSC tissues compared to paired ovarian neoplasm tissues (Figure 3A). The Kaplan-Meier Plotter subset miRpower for Pan-cancer suggested an association between low expression of miR-363 (the precursor of miR-363-3p) and poor OS in 486 ovarian cancer patients. The median OS for patients with low miR-363 expression was notably shorter at 36.8 months compared to 48.37 months in patients with high miR-363 expression (Figure 3B). In OSC patients, upregulation of miR-363-3p correlated with better survival outcomes, as demonstrated by OncomiR data (Figure 3C).

### High expression of ADAM10 was correlated with poor patient survival in biomedical databases

**Figure 4:**
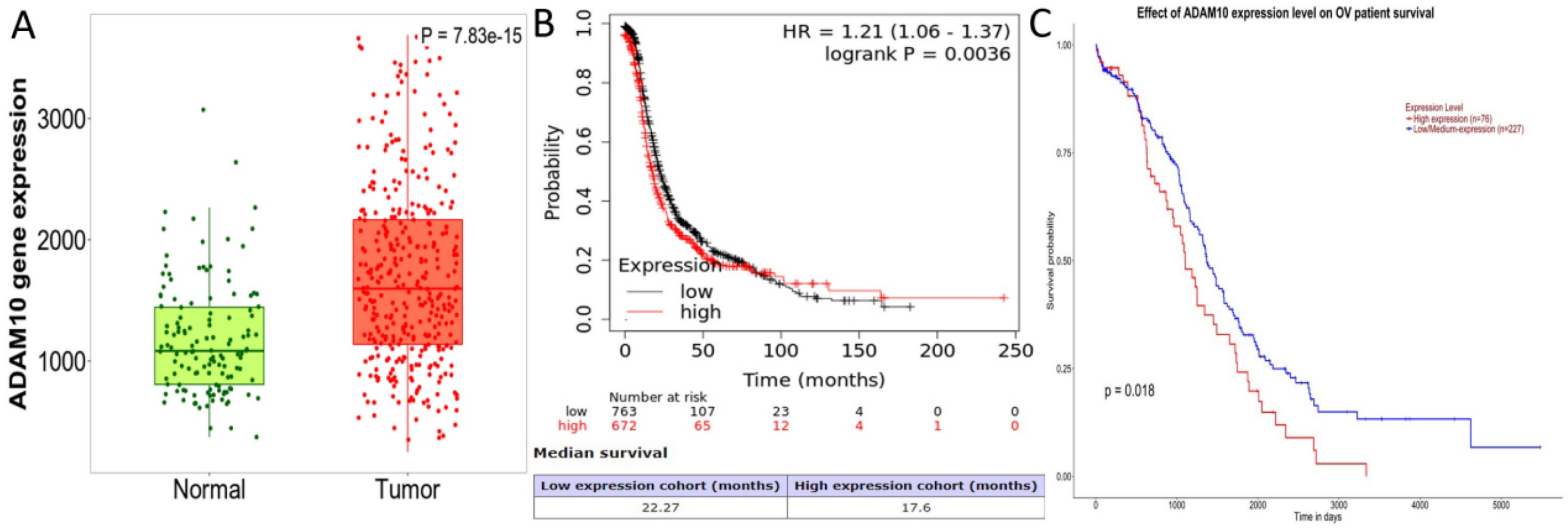
ADAM10 Expression Levels in OSC and their Association with Patient Survival ( A. Comparative analysis of ADAM10 expression in OSC and matched normal ovarian tissues, data derived from the Kaplan-Meier Plotter database; B. Relationship between high ADAM10 expression and poor Progression-Free Survival (PFS) in ovarian cancer, as indicated by the Kaplan-Meier Plotter database; C. Association between elevated ADAM10 expression and poorer survival outcomes in OSC patients, as suggested by the UALCAN database.)

Databases such as TargetScan, microT-CDS, miRDB, miRWalk, and UALCAN predicted that miR-363-3p could bind to the 3 'UTR region of the ADAM10 gene (Figure 2C-D). The Kaplan-Meier Plotter subset TN-plot indicated that ADAM10 expressions in 374 OSC tissues were significantly higher than in 133 paired normal ovarian tissues (Figure 4A). The Kaplan-Meier Plotter subset Ovarian cancer mRNA showed that upregulated ADAM10 expression correlated with poorer PFS in 1436 ovarian cancer patients. Patients with high ADAM10 expression had a median PFS of 17.6 months, which was considerably shorter than the 22.27 months observed in patients with low ADAM10 expression (Figure 4B). The UALCAN subset TCGA Gene also suggested a trend towards poorer survival in OSC patients with high ADAM10 expression (Figure 4C).

### Expressions profiles of PURPL, miR-363-3p and ADAM10 in assessed ovarian tissues

**Figure 5:**
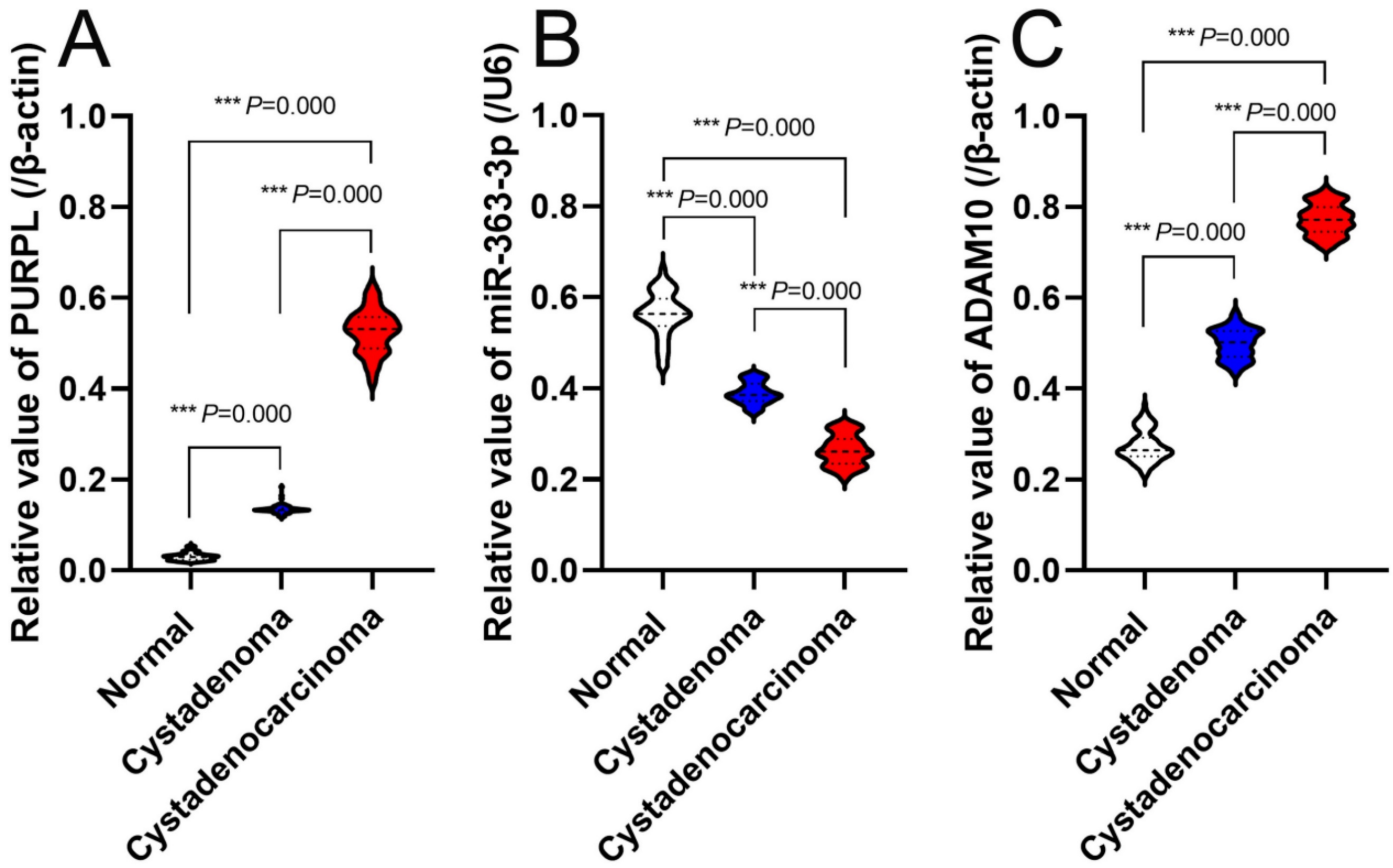
Real-Time PCR-Assessed Expression Profiles of PURPL, miR-363-3p, and ADAM10 in the Assessed Ovarian Tissues.

Expression Profiles of PURPL, miR-363-3p, and ADAM10 in Evaluated Ovarian Tissues Each specimen underwent three independent real time RT-PCR tests, revealing that the expression levels of PURPL in 81 instances of OSC tissues were significantly elevated compared to normal ovarian and ovarian serous cystadenoma tissues (F=7621.000, P<0.0001), as depicted in Figure 5A. Conversely, miR-363-3p expressions in OSC tissues were markedly lower than those in normal ovarian and ovarian serous cystadenoma tissues (F=2323.000, P<0.0001), illustrated in Figure 5B. Furthermore, ADAM10 expression in OSC tissues was significantly higher than in normal ovarian and ovarian serous cystadenoma tissues (F=7747.000, P<0.0001), represented in Figure 5C.

### Expression correlations of PURPL, miR-363-3p and ADAM10 in assessed ovarian tissues

**Figure 6:**
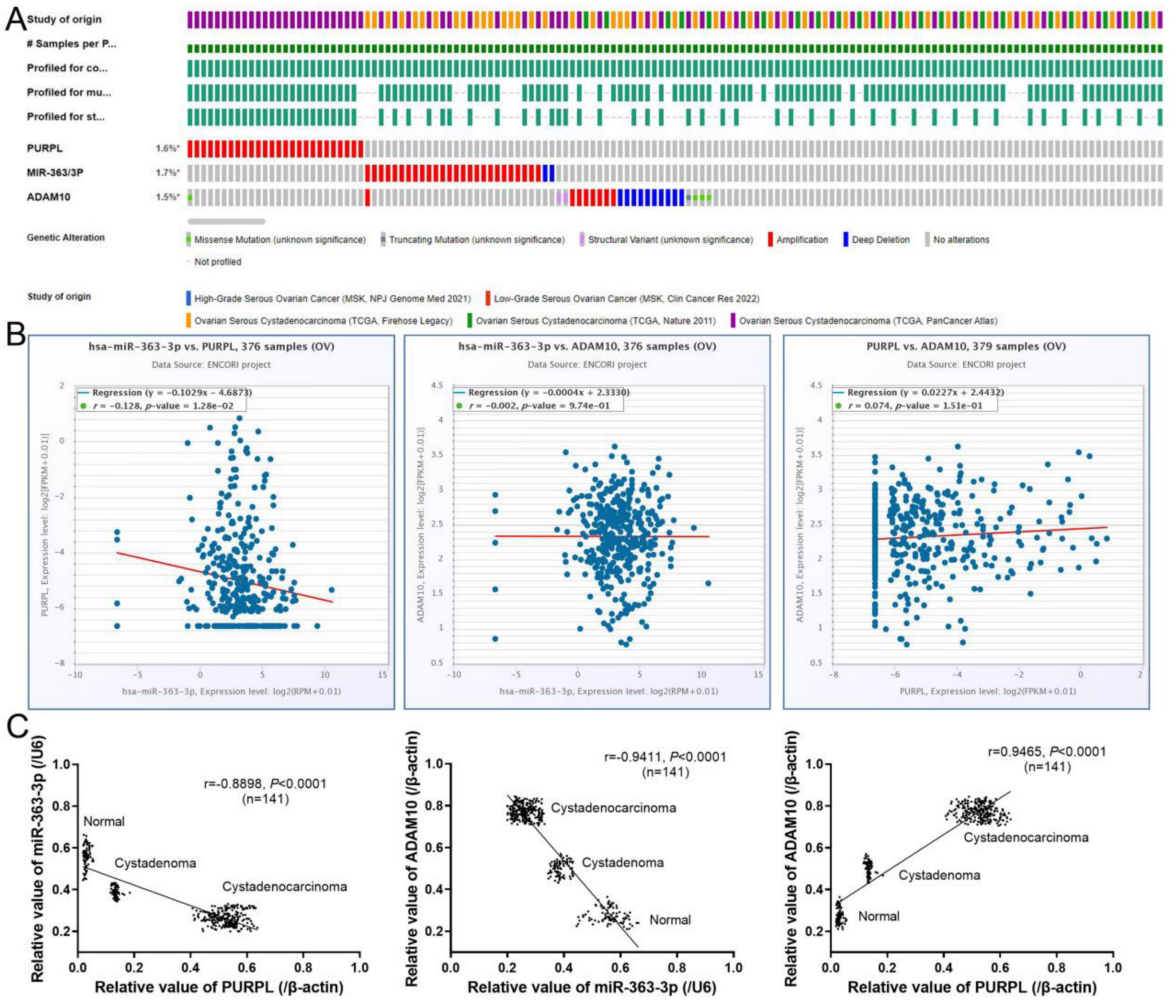
Correlative Expression of PURPL, miR-363-3p, and ADAM10 in the Evaluated Ovarian Tissues.

Correlative Expression of PURPL, miR-363-3p, and ADAM10 in Evaluated Ovarian Tissues Comparative expression patterns of PURPL, miR-363-3p, and ADAM10 were discernible in 1855 samples of 5 OSC tissue cohorts, as queried in the cBioPortal database (Figure 6A). ENCORI database revealed pairwise comparison correlations among PURPL, miR-363-3p, and ADAM10 (Figure 6B). Notably, a statistically significant negative correlation emerged between the expression of PURPL and miR-363-3p in OSC tissues. Additionally, our specimen analysis indicated a significant negative correlation between PURPL and miR-363-3p expression (r = -0.8898, P <0.0001) across all 141 specimens of varying ovarian tissues. Concurrently, a clear negative correlation between miR-363-3p and ADAM10 expression (r = -0.9411, P <0.0001), along with a distinct positive correlation between PURPL and ADAM10 expression (r = 0.9465, P <0.0001), were observed in the same 141 specimens, as displayed in Figure 6C.

### Relations between abnormal expression and clinical parameters of OSC

Associations Between Aberrant Expression and OSC Clinical Parameters For subsequent tissue analyses, the median relative levels of PURPL, miR-363-3p, and ADAM10 in the 81 OSC tissue cases were utilized to differentiate between relatively high (at or above the median expression) and low (below the median expression) expression groups.

Upon individual analyses and comparisons of the current OSC specimens, we discovered that PURPL upregulation and miR-363-3p downregulation were associated with advanced clinical stage and developed lymph node metastasis, respectively. Additionally, elevated ADAM10 expression correlated with advanced clinical stage (Table 2).

**Figure 7:**
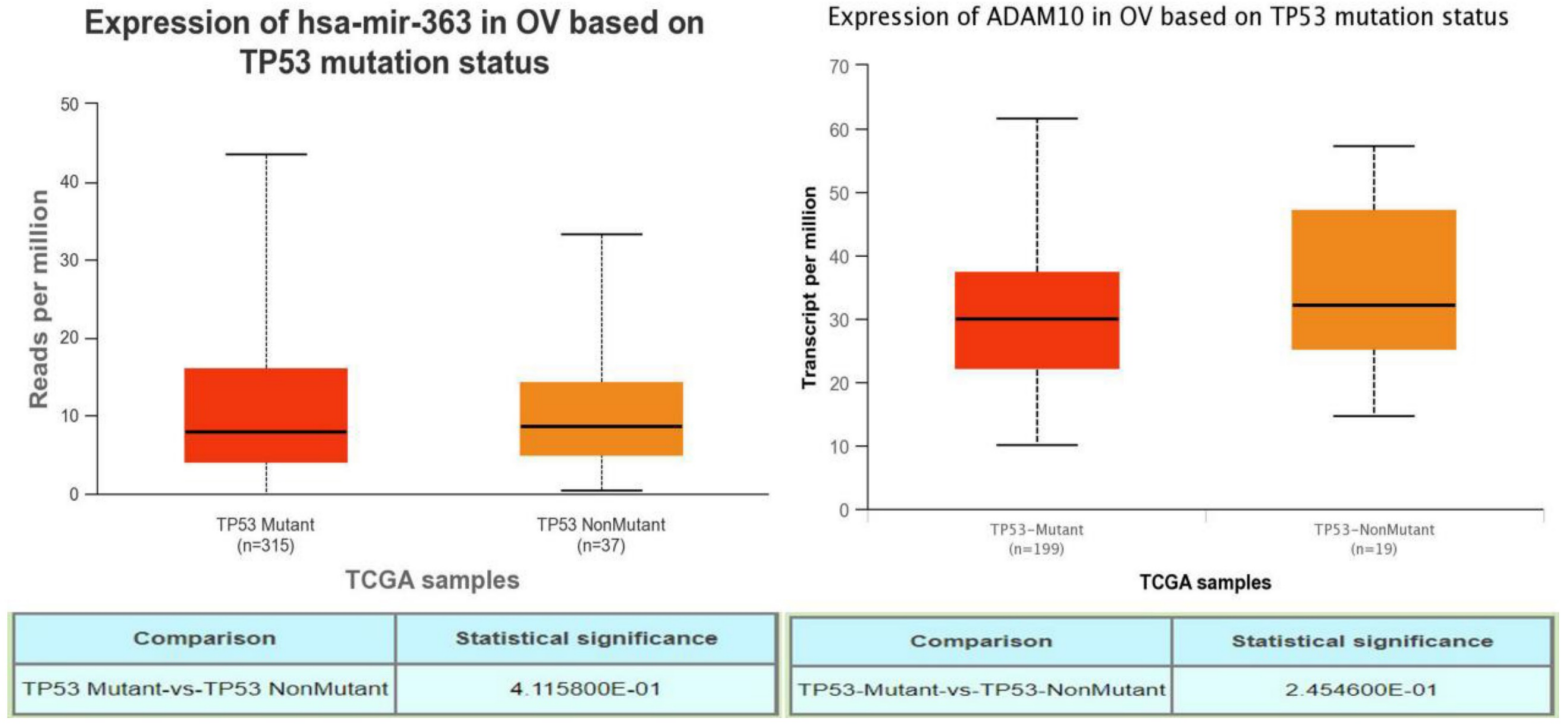
Correlations between miR-363-3p and ADAM10 Expressions and TP53 Mutation in OSC Patients, as Evidenced by the UALCAN Database.

A TP53 mutation is a notable characteristic of high-grade OSC, often leading to poor prognosis. To investigate if abnormal expressions of PURPL, miR-363-3p, and ADAM10 were associated with TP53 mutation in OSC, UALCAN was utilized to explore the relations between expression and TP53 mutation status. While no record of PURPL was indicated in UALCAN, there were no observable correlations between the expressions of miR-363 (precursor of miR-363-3p) or ADAM10 and TP53 gene mutation status (Figure 7). Interestingly, PURPL upregulation was found to be associated with a high TP53 mutation rate in the present tissue study (Table 2).

### Survival predicted values of abnormal expressions of PURPL, miR-363-3p and ADAM10 for the patients with OSC

**Figure 8:**
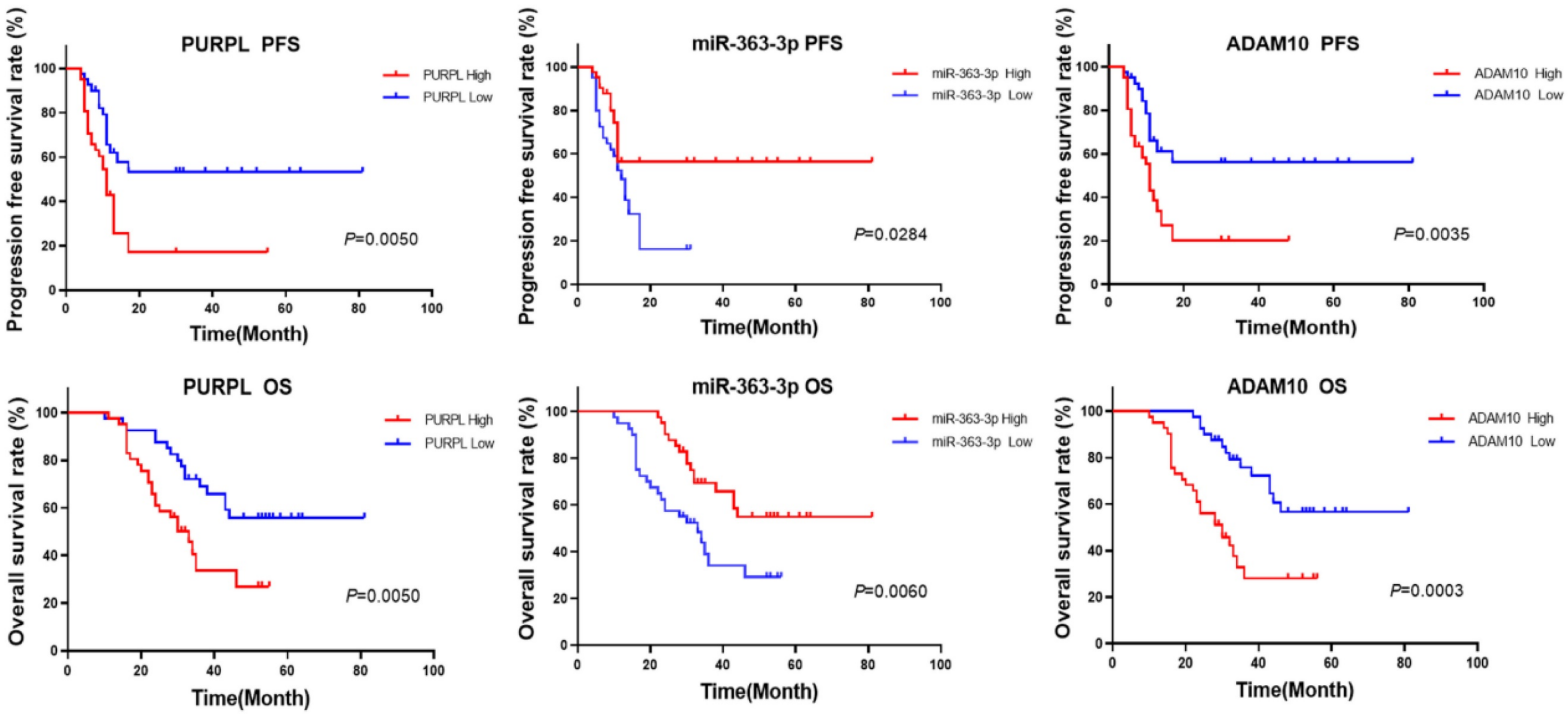
Kaplan-Meier Analysis of the Predictive Values of Abnormal Expressions of PURPL, miR-363-3p, and ADAM10 for Survival in OSC Patients.

The aforementioned database information proposes that abnormal expressions of PURPL, miR-363-3p, and ADAM10 could be correlated with the survival rates of patients with OSC. In the current study, we utilized the median expression as a demarcation point to analyze the relationships between the expressions of PURPL, miR-363-3p, and ADAM10, and the survival of OSC patients. Our findings demonstrated that abnormal expressions of PURPL, miR-363-3p, and ADAM10 were all associated with the survival outcomes of OSC patients. Specifically, overexpression of PURPL, underexpression of miR-363-3p, and overexpression of ADAM10 individually suggested inferior PFS and OS of OSC patients (Figure 8).

**Figure 9:**
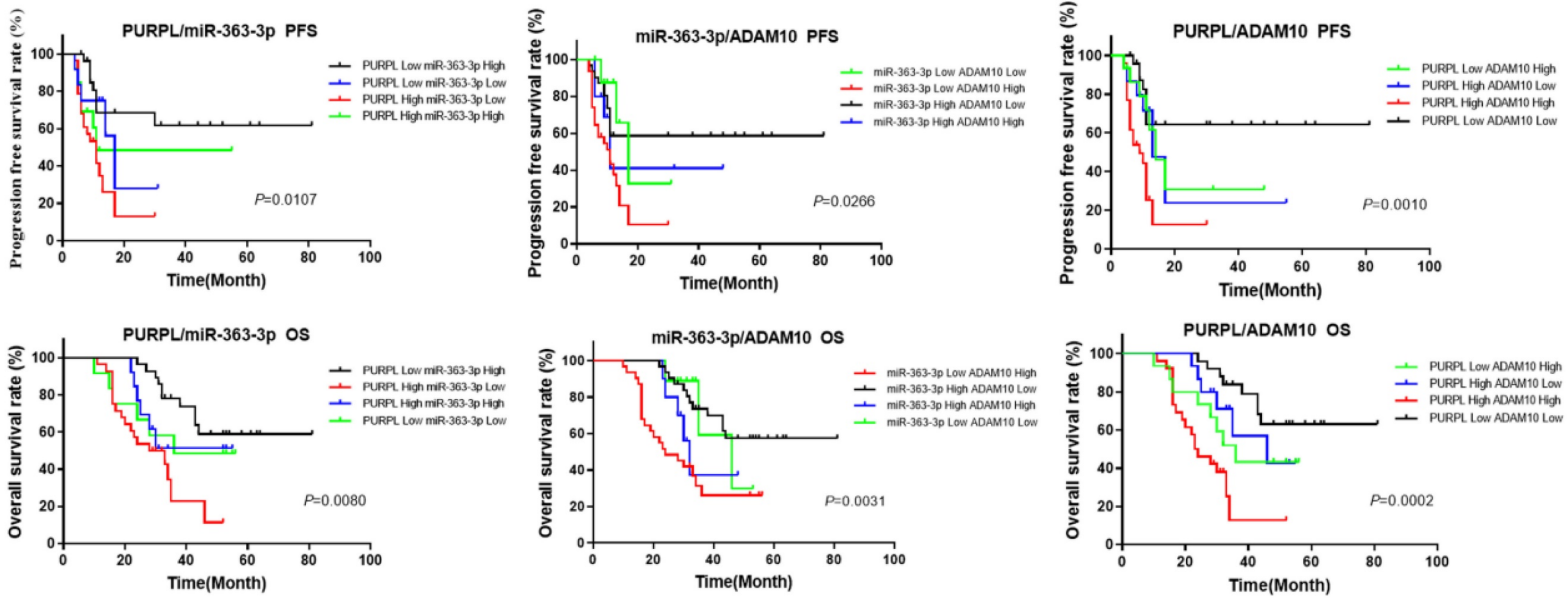
Kaplan-Meier Analysis of the Synergistic Impact of Abnormal Expressions of PURPL, miR-363-3p, and ADAM10 on OSC Survival.

Given that the bioinformatics analysis hinted at potential upstream-downstream targeted regulatory relationships among PURPL, miR-363-3p, and ADAM10, we proceeded to examine the cumulative prognostic value of these three factors in OSC patients. The pairwise analysis of the three factors revealed that OSC patients demonstrating a high level of PURPL and a low level of miR-363-3p, those with a high level of both PURPL and ADAM10, and those with a low level of miR-363-3p and a high level of ADAM10 presented substantially poorer PFS and OS compared to the other patient groups (Figure 9).

## Discussion

Ovarian serous cystadenocarcinoma (OSC), the most prevalent histotype of ovarian cancer, diverges in genomic characteristics from endometrioid, clear cell, and mucinous ovarian cancer. The advent of an array of novel therapeutic agents in recent years, including angiogenesis inhibitors 19,20, PARP inhibitors 21,22, cell cycle checkpoint inhibitors 23,24, and immune checkpoint inhibitors 25,26, has paved the way for innovative approaches to ovarian cancer treatment, offering a glimmer of hope for extending patient survival. It is crucial to underscore that the substantial advancements in these treatment modalities predominantly apply to OSC. In light of the current clinical landscape, OSC remains the deadliest malignancy of the female reproductive system 27,28. Consequently, the exploration of factors exhibiting abnormal expression or those potentially influential in the progression of malignancy remains an unignorable research direction in the treatment or prognostic monitoring of malignant tumors.

It has been well-established that noncoding RNAs, including lncRNA and miRNA, play instrumental roles in human cellular physiology, pathological mechanisms, and carcinogenesis. They can also serve as prognostic markers and therapeutic targets for human diseases, including cancer 29-32. The lncRNA PURPL was first identified as overexpressed and promotive of tumorigenesis in colorectal cancer 33. The subsequent observations reported analogous expression profiles and carcinogenic effects of PURPL in liver cancer 34, gastric cancer 35, and ovarian cancer 10. Extensive literature denotes that miR-363-3p, sponged by various lncRNAs, is implicated in the development and progression of numerous human malignancies, including colorectal cancer, hepatocellular carcinoma, gastric cancer, non-small cell lung cancer, and ovarian cancer 36-40. As a significant member of the disintegrin metalloproteinase family, ADAM10 liberates a variety of membrane-binding proteins, including Notch, E-cadherin, CD44, ErbB receptor HER2, and EGFR, all of which are closely involved in cell malignant proliferation, differentiation, adhesion, and migration. Thus, ADAM10 is regarded as a potential therapeutic target for interventions in human malignancies 41-42.

Our previous research ascertained that PURPL is upregulated in ovarian cancer, and its upregulation correlates with poor Recurrence-Free Survival (RFS) and OS for ovarian cancer patients 10. To further illuminate the molecular mechanisms of the PURPL-regulated network of miRNA and downstream target genes implicated in OSC pathogenesis, we initiated the present study to mine data from open-access medical databases, thereby enriching the understanding of PURPL's molecular functionalities. In OSC specifically, PURPL exhibited an overexpression profile in the Kaplan-Meier Plotter database and suggested an association with poor survival outcomes in the UALCAN database. Among the miRNAs that databases predict could be targeted by PURPL, miR-363-3p was downregulated in OSC and associated with poor patient survival, as indicated by the miRGator, Kaplan-Meier Plotter, and OncomiR databases, respectively. Concerning the genes targeted by miR-363-3p, ADAM10 emerged as a clinically significant marker with abnormally high expression in OSC and was associated with poor prognosis for OSC patients, as evidenced by the Kaplan-Meier Plotter and UALCAN databases.

Database interrogation suggested that the PURPL/miR-363-3p/ADAM10 regulatory axis might be implicated in OSC pathogenesis and correlate with poor prognosis. After obtaining ethical clearance, we procured tissue samples for real time RT-PCR analysis to authenticate the outcomes obtained from the biomedical databases. We identified overexpression of PURPL, underexpression of miR-363-3p, and upregulation of ADAM10 in the OSC tissues enrolled for this study, in contrast to normal ovarian tissues and serous cystadenoma tissues. Furthermore, we found that PURPL expression inversely correlated with miR-363-3p expression, miR-363-3p expression inversely correlated with ADAM10 expression, and PURPL expression positively correlated with ADAM10 expression in the ovarian tissues with varying properties that were studied. Collating the database-predicted target-relationships for PURPL, miR-363-3p, and ADAM10, these findings imply that PURPL, miR-363-3p, and ADAM10 might participate in OSC carcinogenesis, orchestrating an upstream-downstream regulatory axis.

Examining the relationships between aberrant expression and clinical prognosis of OSC patients, we first noted that overexpressions of PURPL and underexpressions of miR-363-3p were associated with a more advanced FIGO stage and the development of lymph node metastasis, while overexpressions of ADAM10 were related to a more advanced FIGO stage. Overexpression of PURPL could attenuate basal p53 levels. PURPL associated with MYBBP1A via the adaptor protein HuR, hindering the formation of a p53-MYBBP1A complex, consequently suppressing the activation and stabilization of basal wild type p53 levels 33. TP53 gene mutation is pervasive in OSC, particularly in high-grade OSC, and is linked with a poor prognosis. In our study, we found a positive relationship between PURPL expression and TP53 mutation. However, miR-363-3p and ADAM10 expressions did not exhibit an association with TP53 mutation. Secondly, the Log-rank analysis projected that upregulation of PURPL, downregulation of miR-363-3p, upregulation of ADAM10 were correlated with OSC recurrence and mortality. In addition, OSC patients presenting with a high level of PURPL and a low level of miR-363-3p, those with a high level of PURPL and ADAM10, and those with a low level of miR-363-3p and high level of ADAM10 were also associated with disease recurrence and death.

## Conclusions

Predicted through biomedical database interrogation and corroborated by tissue analysis, the present study posits that upregulation of PURPL, downregulation of miR-363-3p, and upregulation of ADAM10 are observable in OSC. The aberrant expressions of PURPL, miR-363-3p, and ADAM10 might partake in the pathogenesis of OSC by forming an upstream-downstream regulation mechanism. Furthermore, these abnormal expressions may portend a poor prognosis for OSC.

## Figures and Tables

**Table 1:** Primer sequences for real time RT-PCR

Item	Sequences (5' to 3')
PURPL	Forward: ACACGGGGCTTGAGAAATGA
	Reverse: TCAATCTCCAAAATAGCCGGA
ADAM10	Forward: TTTGGATCCCCACATGATTCTG
	Reverse: GGTTGGCCAGATTCAACAAAAC
β-actin	Forward: CATGTACGTTGCTATCCAGGC
	Reverse: CTCCTTAATGTCACGCACGAT
miR-363-3p	Forward: CGGCGAATTGCACGGTATCCA
	Reverse: CAGTGCAGGGTCCGAGGT
U6	Forward: CTCGCTTCGGCAGCACA
	Reverse: AACGCTTCACGAATTTGCGT

**Table 2:** Associations between PURPL, miR-363-3p, and ADAM10 Expressions in OSC Tissues and the Clinical Parameters of OSC (n, %).

Item	n	PURPL		χ^2^ value	*P* value	miR-363-3p		χ^2^ value	*P* value	ADAM 10		χ^2^ value	*P* value
		High	Low			High	Low			High	Low		
**Age (year)**													
<54	40	21(52.50)	19(47.50)			22(55.00)	18(45.00)			19(47.50)	21(52.50)		
≥54	41	20(48.78)	21(51.22)	0.112	0.738	19(46.34)	22(53.66)	0.607	0.436	22(53.66)	19(46.34)	0.307	0.579
**FIGO stage**													
Ⅰ-Ⅱ	14	2(14.29)	12(85.71)			12(85.71)	2(14.29)			3(21.43)	11(78.57)		
Ⅲ-Ⅳ	67	39(58.21)	28(41.79)	6.118	0.013*	29(43.28)	38(56.72)	8.341	0.004**	38(56.72)	29(43.28)	5.769	0.016*
**Histological grade**													
Low	18	6(33.33)	12(66.67)			12(66.67)	6(33.33)			7(38.89)	11(61.11)		
High	63	35(55.56)	28(44.44)	2.766	0.096	29(46.03)	34(53.97)	2.385	0.123	34(53.97)	29(46.03)	1.274	0.259
**TP53 gene mutation**													
NO	30	8(26.67)	22(73.33)			19(63.33)	11(36.67)			12(40.00)	18(60.00)		
Yes	51	33(64.71)	18(35.29)	10.934	0.001^***^	22(43.14)	29(56.86)	3.082	0.079	29(56.86)	22(43.14)	2.149	0.143
**Lymph node metastasis**													
No	35	13(37.14)	22(62.86)			23(65.71)	12(34.29)			15(42.86)	20(57.14)		
Yes	46	28(60.87)	18(39.13)	4.477	0.034^*^	18(39.13)	28(60.87)	5.620	0.018*	26(56.52)	20(43.47)	1.485	0.223
**Ascites**													
No	25	9(36.00)	16(64.00)			15(60.00)	10(40.00)			10(40.00)	15(60.00)		
Yes	56	32(57.14)	24(42.86)	3.091	0.079	26(46.43)	30(53.57)	1.274	0.259	31(55.36)	25(44.64)	1.631	0.202

## Abbreviations

OSC: ovarian serous cystadenocarcinoma
lncRNAs: long noncoding RNAs
miRNAs: micro noncoding RNAs
PURPL: p53 upregulated regulator of p53 levels
miR-363-3p: microRNA-363-3p
ADAM10: ADAM metallopeptidase domain 10
PFS: Progression free survival
OS: Overall survival

## References

[1] Kiełbowski K, Ptaszyński K, Wójcik J (2023). The role of selected non-coding RNAs in the biology of non-small cell lung cancer. Adv Med Sci.

[2] Schwarzenbach H, Gahan PB (2023). Interplay between LncRNAs and microRNAs in Breast Cancer. Int J Mol Sci.

[3] Shetty A, Venkatesh T, Kabbekodu SP (2022). LncRNA-miRNA-mRNA regulatory axes in endometrial cancer: a comprehensive overview. Arch Gynecol Obstet.

[4] López-Camarillo C, Ruíz-García E, Salinas-Vera YM (2021). Deciphering the Long Non-Coding RNAs and MicroRNAs Coregulation Networks in Ovarian Cancer Development: An Overview. Cells.

[5] Xu Y, Sun Y, Song X (2023). The mechanisms and diagnostic potential of lncRNAs, miRNAs, and their related signaling pathways in cervical cancer. Front Cell Dev Biol.

[6] Han S, Chen X, Huang L (2023). The tumor therapeutic potential of long non-coding RNA delivery and targeting. Acta Pharm Sin B.

[7] Wang H, Meng Q, Qian J (2022). Review: RNA-based diagnostic markers discovery and therapeutic targets development in cancer. Pharmacol Ther.

[8] Seborova K, Vaclavikova R, Rob L (2021). Non-Coding RNAs as Biomarkers of Tumor Progression and Metastatic Spread in Epithelial Ovarian Cancer. Cancers (Basel).

[9] Toden S, Zumwalt TJ, Goel A (2021). Non-coding RNAs and potential therapeutic targeting in cancer. Biochim Biophys Acta Rev Cancer.

[10] Zhang R, He T, Shi H (2021). Disregulations of PURPL and MiR-338-3p Could Serve As Prognosis Biomarkers for Epithelial Ovarian Cancer. J Cancer.

[11] Paraskevopoulou MD, Vlachos IS, Karagkouni D (2016). DIANA-LncBase v2: indexing microRNA targets on non-coding transcripts. Nucleic Acids Res.

[12] Li JH, Liu S, Zhou H (2014). starBase v2.0: decoding miRNA-ceRNA, miRNA-ncRNA and protein-RNA interaction networks from large-scale CLIP-Seq data. Nucleic Acids Res.

[13] Lánczky A, Győrffy B (2021). Web-Based Survival Analysis Tool Tailored for Medical Research (KMplot): Development and Implementation. J Med Internet Res.

[14] Wong NW, Chen Y, Chen S (2018). OncomiR: an online resource for exploring pan-cancer microRNA dysregulation. Bioinformatics.

[15] Garcia DM, Baek D, Shin C (2011). Weak seed-pairing stability and high target-site abundance decrease the proficiency of lsy-6 and other microRNAs. Nat Struct Mol Biol.

[16] Chen Y, Wang X (2020). miRDB: an online database for prediction of functional microRNA targets. Nucleic Acids Res.

[17] Chandrashekar DS, Karthikeyan SK, Korla PK (2022). UALCAN: An update to the integrated cancer data analysis platform. Neoplasia.

[18] Zhang R, Shi H, Ren F (2019). Down-regulation of miR-338-3p and Up-regulation of MACC1 Indicated Poor Prognosis of Epithelial Ovarian Cancer Patients. J Cancer.

[19] Gaitskell K, Rogozińska E, Platt S (2023). Angiogenesis inhibitors for the treatment of epithelial ovarian cancer. Cochrane Database Syst Rev.

[20] Simion L, Rotaru V, Cirimbei C (2023). Analysis of Efficacy-To-Safety Ratio of Angiogenesis-Inhibitors Based Therapies in Ovarian Cancer: A Systematic Review and Meta-Analysis. Diagnostics (Basel).

[21] Tang H, Kulkarni S, Peters C (2023). The Current Status of DNA-Repair-Directed Precision Oncology Strategies in Epithelial Ovarian Cancers. Int J Mol Sci.

[22] Caruso G, Tomao F, Parma G (2023). Poly (ADP-ribose) polymerase inhibitors (PARPi) in ovarian cancer: lessons learned and future directions. Int J Gynecol Cancer.

[23] Gupta N, Huang TT, Horibata S (2022). Cell cycle checkpoints and beyond: Exploiting the ATR/CHK1/WEE1 pathway for the treatment of PARP inhibitor-resistant cancer. Pharmacol Res.

[24] Arend RC, Jackson-Fisher A, Jacobs IA (2021). Ovarian cancer: new strategies and emerging targets for the treatment of patients with advanced disease. Cancer Biol Ther.

[25] Truxova I, Cibula D, Spisek R (2023). Targeting tumor-associated macrophages for successful immunotherapy of ovarian carcinoma. J Immunother Cancer.

[26] Kandalaft LE, Dangaj Laniti D (2022). Immunobiology of high-grade serous ovarian cancer: lessons for clinical translation. Nat Rev Cancer.

[27] Siegel RL, Miller KD, Wagle NS (2023). Cancer statistics, 2023. CA Cancer J Clin.

[28] Sheehy J, Rutledge H, Acharya UR (2023). Gynecological cancer prognosis using machine learning techniques: A systematic review of the last three decades (1990-2022). Artif Intell Med.

[29] Suzuki HI (2023). Roles of MicroRNAs in Disease Biology. JMA J.

[30] Motlagh FM, Kadkhoda S, Motamedrad M (2023). Roles of non-coding RNAs in cell death pathways involved in the treatment of resistance and recurrence of cancer. Pathol Res Pract.

[31] Bayraktar E, Bayraktar R, Oztatlici H (2023). Targeting miRNAs and Other Non-Coding RNAs as a Therapeutic Approach: An Update. Noncoding RNA.

[32] Han S, Chen X, Huang L (2023). The tumor therapeutic potential of long non-coding RNA delivery and targeting. Acta Pharm Sin B.

[33] Li XL, Subramanian M, Jones MF (2017). Long Noncoding RNA PURPL Suppresses Basal p53 Levels and Promotes Tumorigenicity in Colorectal Cancer. Cell Rep.

[34] Fu X, Wang Y, Wu G (2019). Long noncoding RNA PURPL promotes cell proliferation in liver cancer by regulating p53. Mol Med Rep.

[35] Moridi H, Karimi J, Tavilani H (2019). Overexpression of PURPL and downregulation of NONHSAT062994 as potential biomarkers in gastric cancer. Life Sci.

[36] Zhang Y, Peng C, Li J (2022). Long non-coding RNA CCDC144NL-AS1 promotes cell proliferation by regulating the miR-363-3p/GALNT7 axis in colorectal cancer. J Cancer.

[37] Wang J, Tang Q, Lu L (2019). LncRNA OIP5-AS1 interacts with miR-363-3p to contribute to hepatocellular carcinoma progression through up-regulation of SOX4. Gene Ther.

[38] Ma HF, He WW, Wang JJ (2020). Long noncoding RNA LINC00858 promotes the proliferation, migration and invasion of gastric cancer cells via the miR-363-3p/FOXP4 axis. Eur Rev Med Pharmacol Sci.

[39] Jin L, Chen C, Huang L (2021). Long noncoding RNA NR2F1-AS1 stimulates the tumorigenic behavior of non-small cell lung cancer cells by sponging miR-363-3p to increase SOX4. Open Med (Wars).

[40] Lou W, Ding B, Zhong G (2019). Dysregulation of pseudogene/lncRNA-hsa-miR-363-3p-SPOCK2 pathway fuels stage progression of ovarian cancer. Aging (Albany NY).

[41] Moss ML, Stoeck A, Yan W (2008). ADAM10 as a target for anti-cancer therapy. Curr Pharm Biotechnol.

[42] Yan H, Vail ME, Hii L (2022). Preferential Antibody and Drug Conjugate Targeting of the ADAM10 Metalloprotease in Tumours. Cancers (Basel).

